# Neurosteroids mediate and modulate the effects of pro-inflammatory stimulation and toll-like receptors on hippocampal plasticity and learning

**DOI:** 10.1371/journal.pone.0304481

**Published:** 2024-06-14

**Authors:** Yukitoshi Izumi, Kazuko A. O’Dell, Anil G. Cashikar, Steven M. Paul, Douglas F. Covey, Steven J. Mennerick, Charles F. Zorumski

**Affiliations:** 1 Departments of Psychiatry, Washington University School of Medicine, St. Louis, MO, United States of America; 2 The Taylor Family Institute for Innovative Psychiatric Research, Washington University School of Medicine, St. Louis, MO, United States of America; 3 Developmental Biology and Anesthesiology, Washington University School of Medicine, St. Louis, MO, United States of America; 4 Developmental Biology, Washington University School of Medicine, St. Louis, MO, United States of America; Nathan S Kline Institute, UNITED STATES

## Abstract

Pro-inflammatory changes contribute to multiple neuropsychiatric illnesses. Understanding how these changes are involved in illnesses and identifying strategies to alter inflammatory responses offer paths to potentially novel treatments. We previously found that acute pro-inflammatory stimulation with high (μg/ml) lipopolysaccharide (LPS) for 10–15 min dampens long-term potentiation (LTP) in the hippocampus and impairs learning. Effects of LPS involved non-canonical inflammasome signaling but were independent of toll-like receptor 4 (TLR4), a known LPS receptor. Low (ng/ml) LPS also inhibits LTP when administered for 2–4 h, and here we report that this LPS exposure requires TLR4. We also found that effects of low LPS on LTP involve the oxysterol, 25-hydroxycholesterol, akin to high LPS. Effects of high LPS on LTP are blocked by inhibiting synthesis of 5α-reduced neurosteroids, indicating that neurosteroids mediate LTP inhibition. 5α-Neurosteroids also have anti-inflammatory effects, and we found that exogenous allopregnanolone (AlloP), a key 5α-reduced steroid, prevented effects of low but not high LPS on LTP. We also found that activation of TLR2, TLR3 and TLR7 inhibited LTP and that AlloP prevented the effects of TLR2 and TLR7, but not TLR3. The enantiomer of AlloP, a steroid that has anti-inflammatory actions but low activity at GABA_A_ receptors, prevented LTP inhibition by TLR2, TLR3 and TLR7. *In vivo*, both AlloP enantiomers prevented LPS-induced learning defects. These studies indicate that neurosteroids play complex roles in network effects of acute neuroinflammation and have potential importance for development of AlloP analogues as therapeutic agents.

## Introduction

Neuroinflammation contributes to the pathophysiology of neuropsychiatric illnesses and is a therapeutic target of interest. In particular, pro-inflammatory responses underlie changes in cognition, emotion and motivation across illnesses including infections, neurodegenerative disorders, epilepsy and primary psychiatric syndromes such as mood, anxiety, substance use and psychotic disorders [1–4]. These diverse effects make it important to understand how pro-inflammatory signaling alters brain networks and contributes to changes in neuronal function and synaptic plasticity.

In recent studies, we examined the role of microglia and microglial-derived messengers in mediating the effects of acute pro-inflammatory stimulation. For initial studies, we used lipopolysaccharide (LPS), an endotoxin from the cell wall of gram-negative bacteria [5–7] and studied its effects on synaptic function and plasticity in the CA1 region of the rodent hippocampus, a key area in learning and emotional processing. Acute, brief (10–15 min) exposures of hippocampal slices to 1–10 μg/ml LPS have little effect on basal synaptic transmission but disrupt the ability to induce long-term potentiation (LTP). LTP inhibition by this LPS treatment involves microglial activation [8], synthesis of the endogenous oxysterol, 25-hydroxycholesterol (25-HC), and induction of N-methyl-D-aspartate receptor (NMDAR)-dependent metaplasticity [9, 10]. Effects of LPS on LTP are mimicked by exogenous 25-HC and are absent in mice deficient in its synthetic enzyme, cholesterol 25-hydroxylase (Ch25H). Furthermore, *in vivo* LPS produces deficits in learning and memory that are reduced in Ch25H knockout mice [9].

The acute effects of high (μg/ml) LPS were not altered by selective antagonists of toll-like receptor 4 (TLR4), a receptor for LPS, but appeared to involve non-canonical TLR4-independent signaling [9 but see 8]. Here we show that longer (2–4 h) exposures to a low (10 ng/ml) concentration of LPS also inhibit LTP [9] but involve activation of TLR4. Given prior work demonstrating a role for TLR4 in modulating brain function [11, 12], we also explored how TLR4 and other TLRs contribute to pro-inflammatory effects that alter hippocampal synaptic function. For these studies, we focused on the role of the neurosteroid, allopregnanolone (AlloP), based on its role as mediator of other forms of NMDAR-dependent LTP inhibition [13], and its ability to reduce inflammatory responses in other cellular systems [14–16].

## Materials and methods

### Animals

We obtained homozygous Ch25h knockout mice (*Ch25h* KO) backcrossed to C57BL/6J for more than 10 generations [17] from Jackson Laboratories (Cat# 016263). C57BL/6J mice (Cat# 000664, Jackson Labs, Bar Harbor ME) were used as wild type controls. Sprague-Dawley albino rats came from Harlan Laboratories (Indianapolis IN). Animals were housed in approved facilities. Animal use was in accord with NIH guidelines and approved by the Washington University Institutional Animal Care and Use Committee (IACUC, Protocol Numbers: 22–0220, 22–0228 and 22–0344). All efforts were made to minimize pain and alleviate animal suffering.

### Hippocampal slice preparation

Hippocampal slices were prepared from postnatal day (P) 30–32 male albino rats or mice using published methods [9, 18, 19]. For slice preparation, animals were fully anesthetized with isoflurane and decapitated by guillotine. Hippocampi were dissected and pinned on an agar base in artificial cerebrospinal fluid (ACSF) containing (in mM): 124 NaCl, 5 KCl, 2 MgSO_4_, 2 CaCl_2_, 1.25 NaH_2_PO_4_, 22 NaHCO_3_, 10 glucose, gassed with 95% O_2_-5% CO_2_ at 4–6°C. The dorsal two-thirds of the hippocampus was cut into 500 μm slices using a rotary slicer and maintained in ACSF at 30°C for at least 2 hours before experiments to allow sustained pre-incubation with TLR agonists and neuroactive steroids. Slices were pre-incubated on mesh in a 10 ml gassed beaker. Other drugs, if applied, were introduced 30 min after neurosteroid pre-incubation in the same beaker.

### Hippocampal slice physiology

At the time of study, a single slice was transferred to a submersion-recording chamber and perfused with 30°C ACSF at 2 ml/min. Extracellular recordings were obtained from the apical dendritic region of area CA1 to monitor field excitatory postsynaptic potentials (EPSPs). EPSPs were evoked using a bipolar stimulating electrode once per minute using 0.1 ms constant current pulses to the Schaffer collateral pathway. Stimulus intensity was half-maximal according to baseline input-output (IO) curves. LTP was induced using a single 100 Hz x 1 s high frequency (tetanic) stimulation (HFS). IO curves were repeated 60 min following HFS administration and were the primary measure of synaptic change in comparison to baseline. For display in summary figures, responses are typically shown at 5 min intervals for clarity.

### Behavioral studies

P30 rats were tested for memory acquisition in a one-trial inhibitory avoidance learning task that is associated with hippocampal LTP [9, 18, 20, 21]. The testing apparatus contained two chambers. One chamber was lit and the other was kept dark; both compartments included a floor of stainless steel rods (4 mm diameter, spaced 10 mm apart) through which an electrical shock was delivered in the dark chamber (12 x 20 x 16 cm). The lit compartment (30 x 20 x 16 cm) was illuminated with four 13 W lights at a light intensity of 1000 lux; light intensity in the dark chamber was < 10 lux. On the first day of testing, rats were placed in the lit chamber and allowed to explore the apparatus by freely moving between chambers for 10 min. One group of rats received a single injection of AlloP (3 mg/kg intraperitoneally, ip), the enantiomer of AlloP (3 mg/kg ip) or vehicle (DMSO). One hour later, rats were administered LPS (1 mg/kg ip), which was given 1 hour prior to training. Another group of rats received a single injection of the 5α-reductase inhibitor, finasteride (3 mg/kg ip) or vehicle (beta-cyclodextrin, CDX). Two hours later, rats were administered LPS (1 mg/kg ip), which was given 1 hour prior to training. For training, rats were initially placed in the lit chamber. When they completely entered the dark chamber, they were administered a single foot shock. On the second day of testing, rats were placed in the lit chamber without any drug treatment and the latency to enter the dark compartment was recorded over a 300 s trial. The doses of AlloP, *ent*-AlloP and finasteride were determined in preliminary experiments and were based on lack of sedation over the time course of the behavioral studies. Pain and suffering were minimized by removing rats from the testing apparatus as soon as they completed the 300 sec exposure to the chamber. Animals were subsequently sacrificed by decapitation under isoflurane anesthesia.

### Chemicals

Salts, LPS and imiquimod were purchased from Millipore Sigma (St. Louis MO). Other agents were from Tocris Biosciences (Bristol UK, TAK-242, Poly I:C and Pam3CSK4), Innaxon Biosciences (Tewksbury UK, IAXO-102), InVivogen (San Diego CA, LPS-RS) and Steroloids (Newport RI, AlloP and finasteride). The enantiomer of AlloP (*ent*-AlloP) [22] was synthesized using methods described previously [23]. Concentrations of agents used in these experiments are based on published studies and our experience with the compounds. These concentrations were selected to have minimal effects on basal transmission in the CA1 region.

### Data collection & analysis

Experiments were performed and analyzed using pClamp software (Molecular Devices, Union City CA). Results in the text are expressed as mean ± SEM, and physiological results are based on analysis of IO curves obtained at baseline and 60 min following HFS. EPSPs were normalized to baseline recordings (taken as 100%). Statistical comparisons were based on IO curves at baseline and 60 minutes following HFS based on changes in the maximal rising slope of EPSPs evoked by 50% maximal stimuli, with p < 0.05 considered significant. A two-tailed unpaired Student’s t-test was used for most comparisons between groups. When appropriate, paired t-tests were used. Numbers reported in the text are the number (N) of animals studied in a condition. For *in vivo* behavioral studies, data were analyzed using Dunn’s Multiple Comparison Test after Kruskal-Wallis test. Statistics were performed using commercial software (SigmaStat, Systat Software, Inc., Richmond City, CA). Data in figures represent continuous monitoring of responses at constant stimulus amplitude and thus may differ from numerical results described in the text, which are based on analysis of IO curves.

## Results

### Effects of low LPS on hippocampal plasticity involve TLR4 and 25-hydroxycholesterol

**Fig 1:**
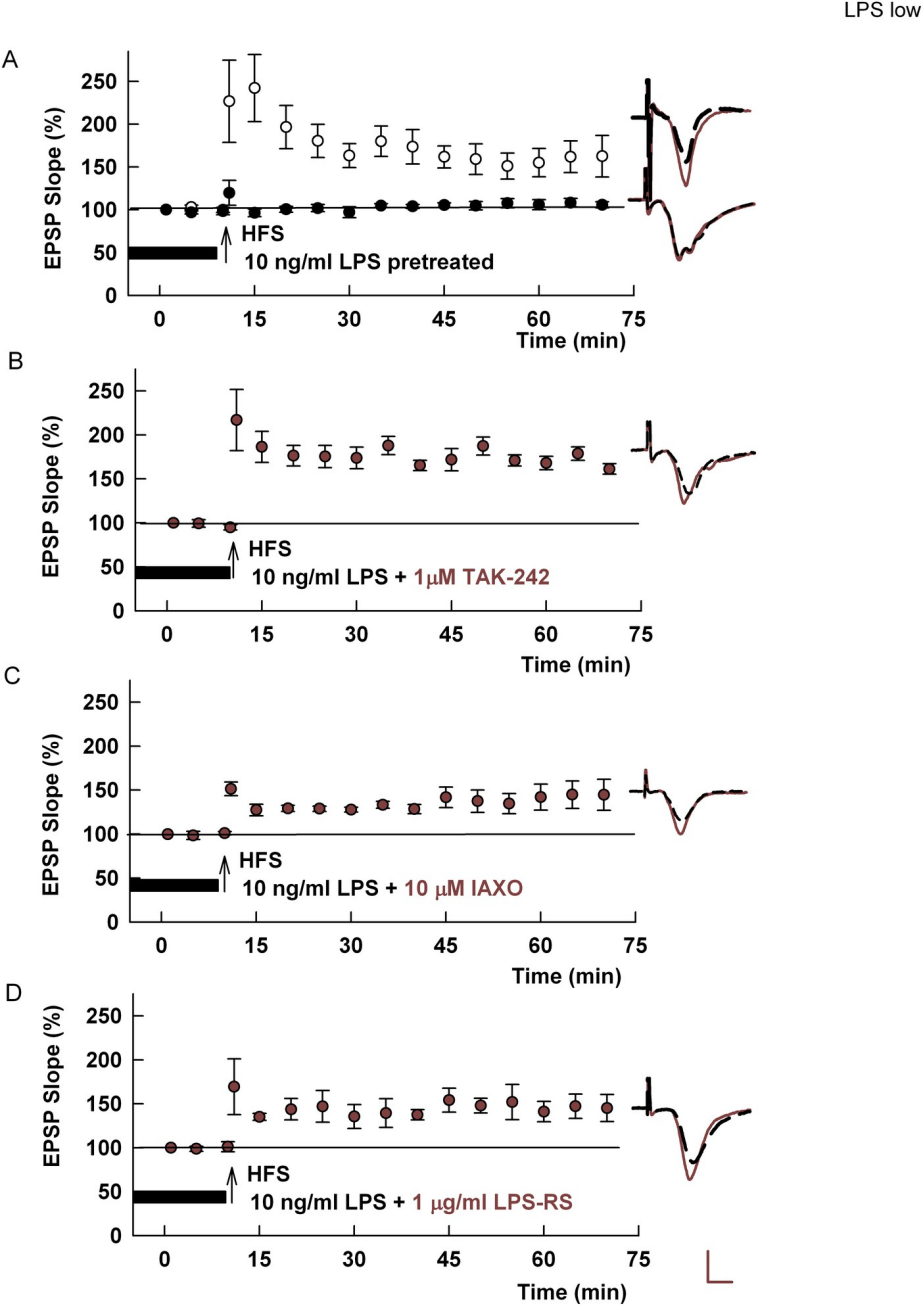
A low (10 ng/ml) concentration of LPS inhibits CA1 LTP via activation of TLR4. A. The graph shows the time course of changes in EPSPs and the ability of a single HFS (arrow) to induce robust LTP (white circles). Incubation of slices with 10 ng/ml LPS for 2–4 hours prior to study completely inhibits LTP (black circles) as shown previously [9] (p = 0.0007 vs. control LTP). B, C, D. Co-treatment of hippocampal slices with three different TLR4 inhibitors, TAK-242 (B) (p = 0.0006 vs. LPS alone); IAXO-102 (C) (p = 0.0031 vs. LPS alone); and LPS-RS (D) (p = 0.0008 vs. LPS alone), prevented the effects of LPS. Traces in this and other figures show representative EPSPs during baseline (dashed traces) and 60 min following HFS (solid traces). Calibration: 1 mV, 5ms.

In prior studies, the adverse effects of high (1–10 μg/ml) LPS administered for 10–15 min just prior to HFS on LTP induction were independent of TLR4 activation based on the insensitivity to two selective TLR4 antagonists [9]. Yet in other studies using a different pro-inflammatory stimulus, the same TLR4 antagonists were effective [24]. Effects of acute μg/ml LPS on LTP were inhibited by LPS-RS, an LPS antagonist that inhibits both TLR4 and non-TLR4 inflammatory signaling [3, 25]. Given that LPS is a known TLR4 agonist [6, 7], we first examined whether the effects of lower concentrations of LPS (ng/ml) engage TLR4 when administered for 2–4 h before tetanic stimulation to inhibit LTP. As we showed previously [9], 10 ng/ml LPS administered by pre-incubation for 2–4 hours completely inhibited LTP induction in the CA1 region (Control LTP: 156.0 ± 8.6% of baseline responses measured 60 min following HFS, N = 7; LPS: 100.9 ± 5.7% of baseline, N = 5, Fig 1A). In contrast to high (μg/ml) concentrations of LPS, we found that the effects of 10 ng/ml LPS were completely blocked by the selective TLR4 antagonists, TAK-242 [26, 27] and IAXO-102 [28]. In slices in which 10 ng/ml LPS was co-administered with 1 μM TAK-242, LTP was readily induced (154.7 ± 7.9%, N = 5, Fig 1B). Similar effects were observed with 10 μM IAXO, another selective TLR4 antagonist (161.4 ± 13.3%, N = 5, Fig 1C). Additionally, the broader spectrum LPS antagonist, LPS-RS (1 μg/ml) prevented the effects of low LPS (145.9 ± 6.5%, N = 5, Fig 1D). In subsequent experiments, we focused on the effects of various treatments on TLR4-dependent LPS, except as noted.

**Fig 2:**
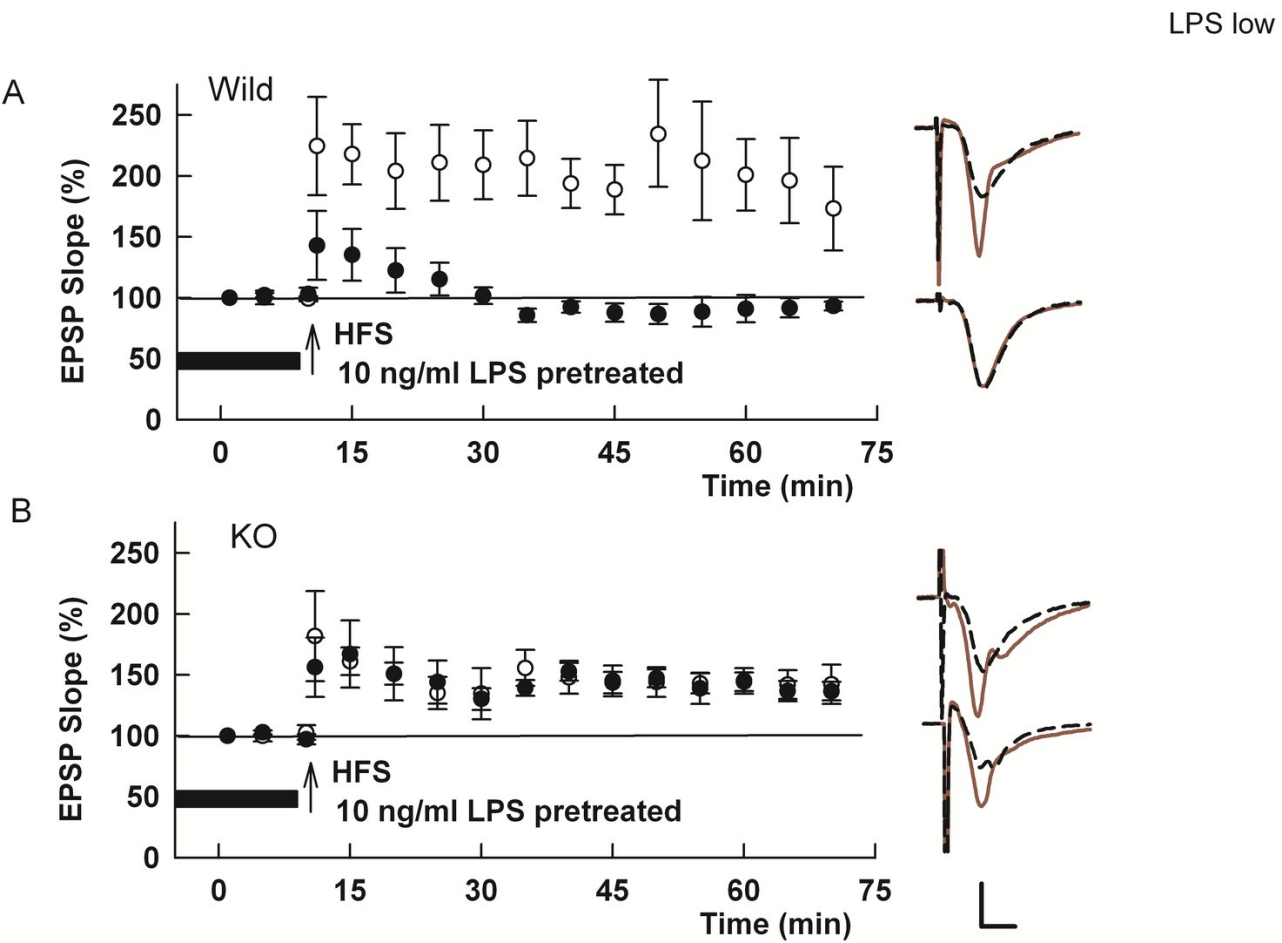
LTP inhibition by low LPS involves the oxysterol, 25-HC. A. In slices from wild type mice, a single HFS readily induces LTP (white circles). LTP in wild type slices is blocked by incubation with 10 ng/ml LPS x 2–4 hours (black circles) (p = 0.0132 vs. control LTP), consistent with our previous observations using brief high (μg/ml) LPS [9]. B. HFS readily induced LTP in slices from *Ch25h* KO mice (white circles) and LPS failed to block LTP in these slices (black circles) (p = 0.5273 vs. control LTP). Traces show EPSPs as in Fig 1. Calibration 1 mV, 5 ms.

We previously found that LTP inhibition by high (μg/ml) LPS involves the endogenous oxysterol, 25-hydroxycholesterol (25-HC), and that exogenous 25-HC mimics effects of LPS on LTP [9]. These latter observations prompted us to examine whether a low concentration of LPS also requires oxysterol synthesis. We tested this using mice with constitutive deletion of cholesterol 25-hydroxylase (Ch25H), the key synthetic enzyme for 25-HC that is expressed largely if not exclusively in microglia [9, 29–31]. In wild type (WT) mice, 2–4 hour administration of 10 ng/ml LPS completely suppressed LTP induction (Control LTP in WT slices: 139.1 ± 11.5% of baseline, N = 5; LPS: 94.3 ± 8.2%, N = 5; Fig 2A). In Ch25H-deficient slices, LPS had no effect on LTP induction (Control LTP in KO slices: 169.2 ± 18.0%, N = 5; KO + LPS: 156.2 ± 7.9%, N = 5; Fig 2B).

### Effects of LPS on hippocampal plasticity are mediated by 5α-reduced neurosteroids

**Fig 3:**
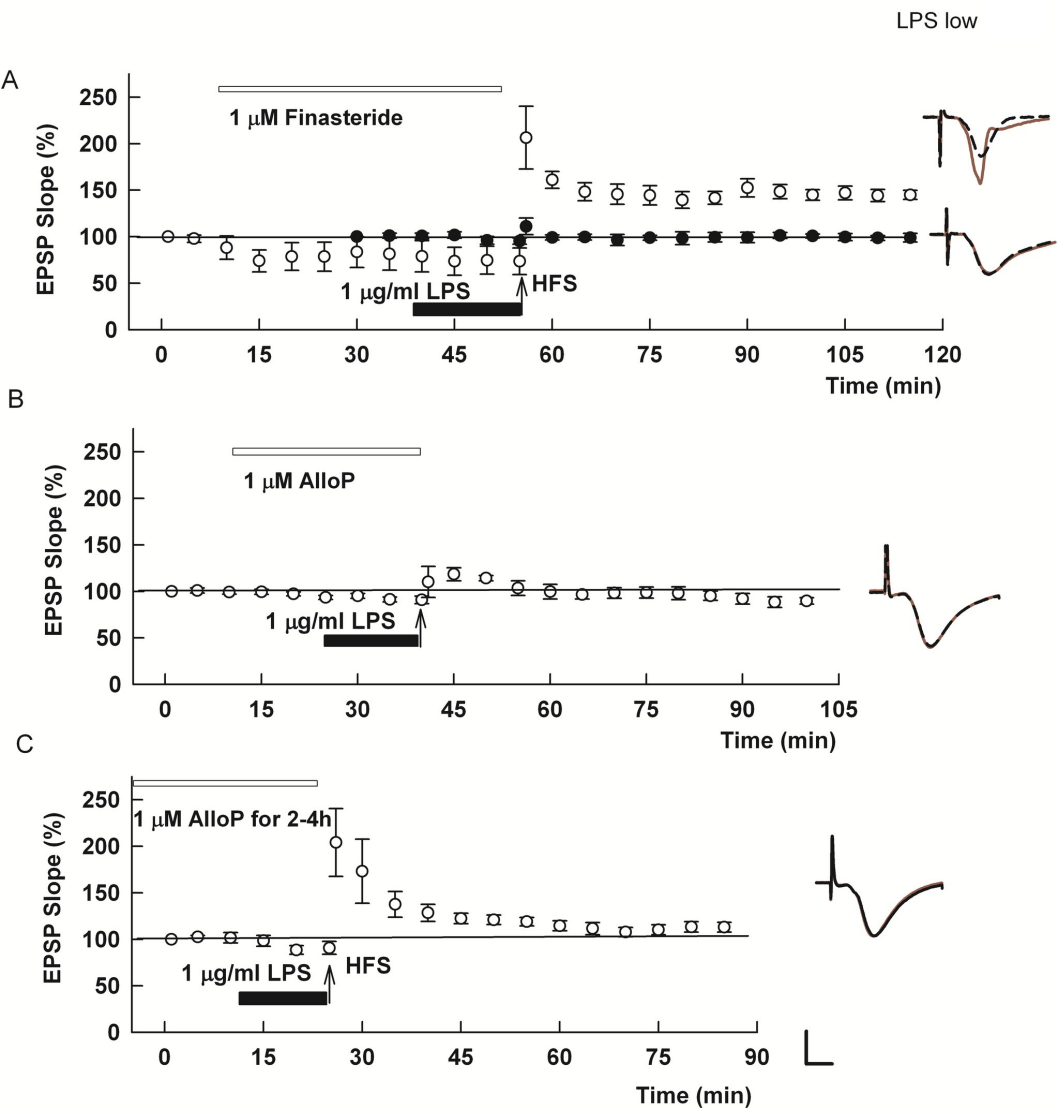
Adverse effects of acute high (1 μg/ml) LPS on LTP involve 5α-reduced neurosteroids, but are not prevented by the neurosteroid, AlloP, alone. A. In the presence of 1 μg/ml LPS administered for 15 min prior to HFS (black bar), CA1 LTP is completely inhibited as we showed previously (black circles) [9]. This LTP inhibition is prevented by the 5α-reductase inhibitor, finasteride (white circles) (p = 0.0038 vs. LPS alone). B,C. Pretreatment with 1 μM exogenous AlloP for 15 min before and during 1 μg/ml LPS failed to promote LTP (p = 0.0937 vs. LPS alone) vs. LPS alone (B) as did 2–4 h preincubation with AlloP (p = 0.2503 vs. LPS alone) (C). Calibration: 1 mV, 5 ms.

In our prior study with high concentrations of LPS, effects on LTP were inhibited by an NMDAR antagonist administered during LPS exposure (Izumi et al., 2021 [9]) and involved NMDAR-dependent metaplasticity that is associated with activation of cellular stress responses [13, 20]. In turn, cellular stress responses promote cholesterol mobilization and synthesis of endogenous neurosteroids, including pregnenolone, a precursor to AlloP [32], as a homeostatic protective mechanism [13, 33]. In several other models of NMDAR-dependent neuronal stress, we found that endogenous 5-alpha reduced neurosteroids such as AlloP contribute to acute inhibition of hippocampal LTP [13, 34]. These observations prompted us to examine whether the effects of LPS are altered by inhibition of 5-alpha reductase (5AR), a key enzyme in AlloP synthesis. In pilot experiments, incubation of slices with the 5AR inhibitor, finasteride, for 2–4 hours to match the duration of low LPS exposure disrupted LTP on its own. Thus, we pursued experiments with 5AR inhibition using 1 μg/ml LPS x 15 min to limit the duration of exposure of slices to 5AR inhibition. We found that 1 μM finasteride prevented the effects of LPS when administered prior to and during LPS administration (LPS: 96.2 ± 5.9% of baseline, N = 5; LPS + finasteride: 155.1 ± 13.4%, N = 5; Fig 3A).

### Allopregnanolone prevents effects of TLR4 activation on hippocampal plasticity

The ability of finasteride to prevent LTP inhibition by LPS is consistent with the ability of LPS to promote cellular stress [9, 13] but differs from other reports indicating that AlloP and similar neurosteroids have anti-inflammatory properties mediated in part by inhibition of TLR4 signaling [14, 15, 35]. These apparent contradictions prompted us to examine the effects of pretreatment of slices with AlloP prior to and during LPS exposure. In initial experiments, we examined whether pre-treatment of slices with AlloP altered acute LTP inhibition by 1 μg/ml (high) LPS. We found that neither acute (15 min prior to and during LPS) nor prolonged administration of 1 μM AlloP (3 hour pretreatment before and during LPS) altered LTP block by high LPS (acute AlloP: 92.8 ± 2.8%, N = 5, Fig 3B; prolonged AlloP: 110.4 ± 4.9%, N = 5, Fig 3C).

**Fig 4:**
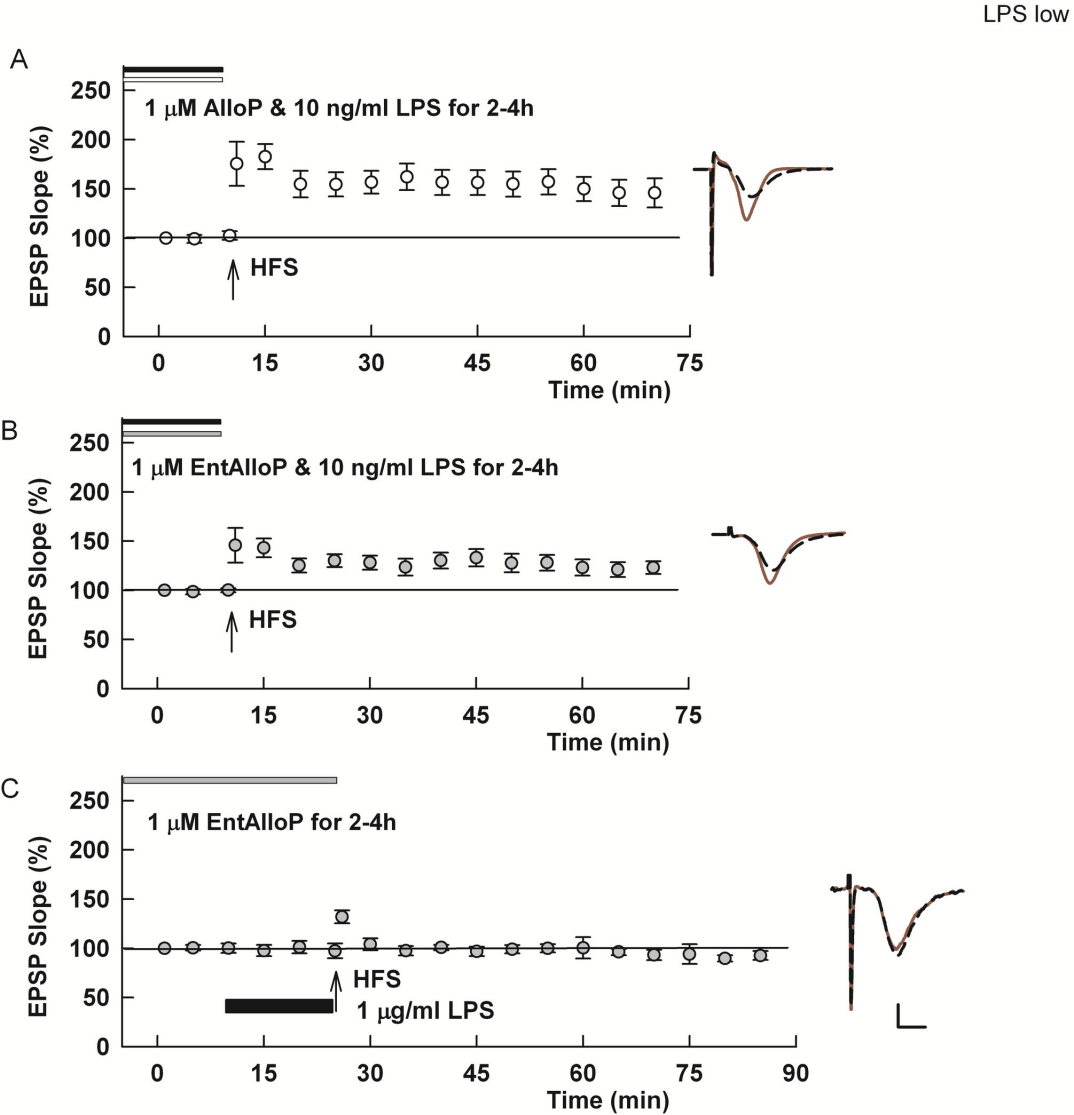
In contrast to high LPS, the effects of low LPS on LTP are prevented by preincubation with AlloP. A. In slices preincubated with 1 μM AlloP plus 10 ng/ml LPS, robust LTP is readily induced by a single HFS (p = 0.0026 vs. LPS alone). B. At 1 μM, the unnatural enantiomer of AlloP had variable effects on LTP inhibition by low LPS but allowed LTP in 6 of 10 slices as described in the text (p = 0.0554 vs LPS alone, N = 10). C. *ent*-AlloP, akin to *nat*-AlloP, failed to alter LTP inhibition by high LPS (p = 0.7929 vs. LPS alone). Traces show representative EPSPs. Calibration: 1 mV, 5 ms.

In contrast to results with high LPS, we found that 1 μM AlloP administered prior to and during 10 ng/ml LPS administration completely prevented the adverse effects of LPS on LTP induction (153.6 ± 10.6% of baseline, N = 6, Fig 4A). Effects of pregnane neurosteroids such as AlloP on GABA_A_Rs are highly enantioselective [22, 36] while neuroprotective and anti-inflammatory effects show no enantioselectivity [36–39]. Thus, we also examined whether the enantiomer of AlloP (*ent*-AlloP) alters the effects of LPS. In hippocampal slices, 1 μM *ent*-AlloP administered before and during 10 ng/ml LPS had variable effects on LTP inhibition (124.2 ± 7.2% of baseline, N = 10; Fig 4B). Six of 10 slices treated with *ent*-AlloP showed LTP >15% increase from baseline 60 min following HFS (range: 116.3% to 169.3%), while the other 4 slices showed changes < 10% of baseline (range: 99.5% to 108.1%). The effects of natural AlloP were significantly greater than its unnatural enantiomer (p = 0.0323). Akin to natural AlloP, preincubation with *ent*-AlloP for 2–4 hours had no effect on LTP inhibition by 1 μg/ml LPS (100.8 ± 3.9% of baseline, N = 4; Fig 4C).

### Allopregnanolone enantiomers modulate hippocampal effects of TLR2, TLR3 and TLR7

**Fig 5:**
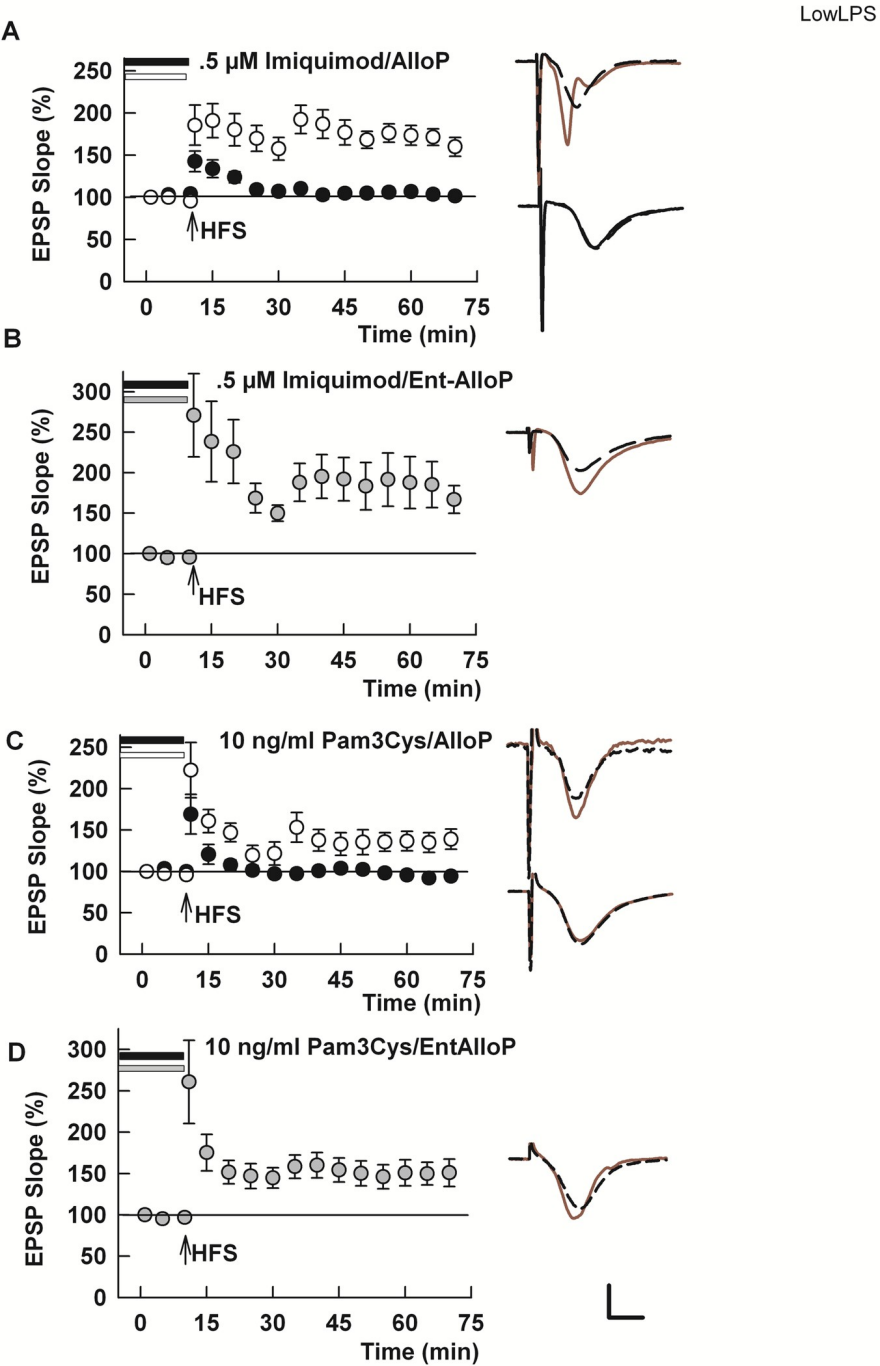
The enantiomers of AlloP prevent the effects of stimulation of TLR7 and TLR2. A. In slices preincubated for 3–4 hours with 0.5 μM imiquimod, an agonist of endosomal TLR7/8 that signals via the adaptor protein MyD88, LTP is completely inhibited (black circles) (p = 0.0003 vs. control LTP). AlloP co-incubation with imiquimod allowed robust LTP (white circles) (p = 0.0119 vs. imiquimod). B. Similar to *nat*-AlloP, *ent*-AlloP prevent the effects of imiquimod on LTP (gray circles and bar) (p = 0.0012 vs. imiquimod). C. Preincubation of slices with 10 ng/ml Pam3Cys, an agonist of TLR2 completely inhibits CA1 LTP (black circles) (p = 0.0003 vs. control LTP), and AlloP prevented the LTP inhibition (white circles) (p = 0.0001 vs. Pam3Cys alone). D. *ent*-AlloP also prevented LTP inhibition by Pam3Cys (gray circles) (p = 0.0024 vs. Pam3Cys alone; p = 0.0398 vs natural AlloP). Calibration: 1 mV, 5 ms.

TLR4 stimulates inflammatory responses sequentially via receptors expressed on plasma membranes and endosomes using the adaptor proteins MyD88 and TRIF-TRAM, respectively [40]. In addition to inhibiting TLR4-mediated inflammatory signaling, AlloP inhibits other MyD88-dependent TLRs, including TLR7 [15], a receptor that signals exclusively from endosomes [41, 42]. Thus, we examined whether activation of TLR7 using the selective agonist imiquimod [43, 44] alters LTP induction and the effects of NAS. When administered for 2–4 hours prior to HFS, imiquimod (0.5 μM), like LPS, completely inhibited LTP induction (93.4 ± 5.9%, N = 5, Fig 5A). Consistent with results showing inhibition of TLR7 by AlloP in a macrophage cell line [15], LTP inhibition by imiquimod was prevented by treatment of slices with 1 μM AlloP (139.9 ± 13.1%, N = 5; Fig 5A). In contrast to TLR4 activation by LPS where *ent*-AlloP had variable effects, we found that 1 μM *ent*-AlloP, akin to its natural analogue, also completely prevented the inhibitory effects of imiquimod on LTP (167.6 ± 14.0, N = 5; p = 0.0012 vs. imiquimod; Fig 5B). Effects of the AlloP enantiomers on imiquimod-induced LTP inhibition did not differ significantly (p = 0.1865).

We also examined effects of the AlloP enantiomers against activation of TLR2. TLR2 interacts with TLR1 to signal from both the plasma membrane and endosomes and uses MyD88 to signal from plasma membranes and several adapters to signal from endosomes [45–47]. We found that the TLR2 agonist Pam3Cys [15] inhibited LTP induction when administered at 10 ng/ml for 3–4 hours (97.1 ± 3.8%, N = 5; Fig 5C). TLR2-mediated LTP inhibition was prevented by AlloP (125.5 ± 2.5% of baseline, N = 6; Fig 5C). The AlloP enantiomer also effectively prevented the effects of Pam3Cys (152.8 ± 12.2%, N = 5; Fig 5D), with the unnatural enantiomer showing a greater effect than AlloP.

**Fig 6:**
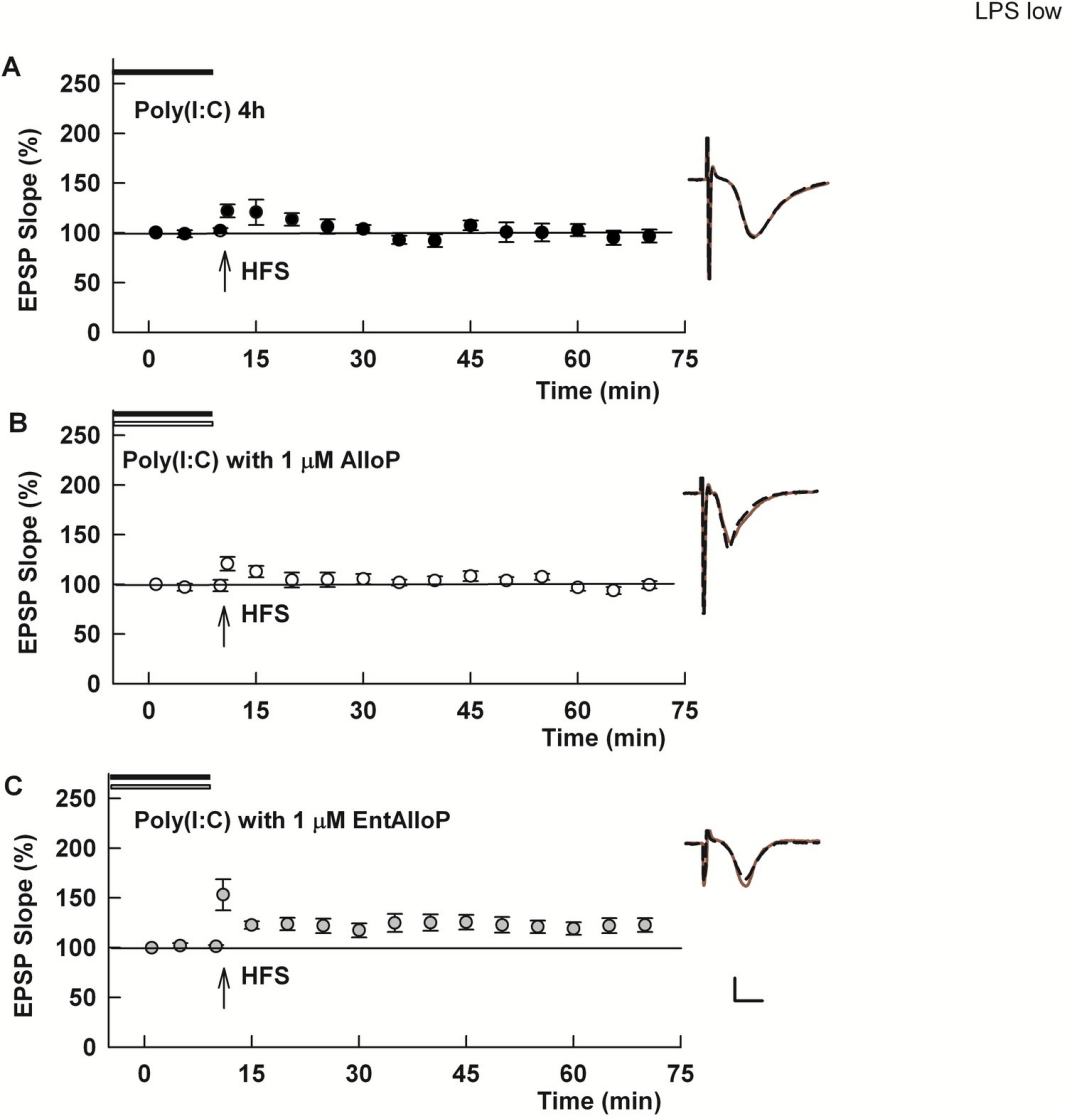
Stimulation of TLR3, a pattern recognition receptor that signals from endosomes in a MyD88-independent fashion, shows reverse AlloP enantioselectivity. A. In slices preincubated with the TLR3 agonist Poly I:C (25 μg/ml) for 3–4 hours, LTP is completely inhibited (p = 0.0005 vs. control LTP). B. Co-incubation of slices with *nat*-AlloP failed to overcome LTP inhibition (p = 0.2228 vs. Poly(I:C) alone) C. In contrast, incubation of slices with *ent*-AlloP allowed small but persisting potentiation following HFS in the presence of Poly I:C (p = 0.0117 vs. Poly(I:C) alone). The degree of potentiation in the presence of Poly I:C and *ent*-AlloP was less than control LTP in naïve slices (p = 0.0009). Calibration 1 mV, 5 ms.

The TLRs outlined above signal at least In part via the MyD88 adaptor protein. In contrast, TLR3 signals exclusively from endosomes via TRIF, a form of signaling shared by TLR2 and TLR4 when they are activated on endosomes. TLR3, however, acts independently of MyD88 unlike the other two TLRs [11, 15]. Previously Balan and colleagues [15] found that cytokine production by TLR3 in a macrophage cell line was not altered by AlloP, in contrast to the TLRs that signal via MyD88. These observations prompted us to examine effects of TLR3 activation on LTP using the agonist Poly(I:C). We found that slices incubated with 25 μg/ml Poly(I:C) for 4 hours showed complete LTP inhibition (103.1 ± 1.9% of baseline, N = 5; Fig 6A). Consistent with Balan et al. [15], we found that AlloP was unable to prevent the effects of Poly(I:C) (96.4 ± 4.7%, N = 5; Fig 6B). In contrast, *ent*-AlloP variably but significantly permitted synaptic enhancement in the presence of the TLR3 agonist (115.8 ± 3.2% of baseline, N = 7, range of EPSP changes = 106.2 to 127.6% of baseline, Fig 6C). The degree of potentiation in the presence of Poly I:C plus *ent*-AlloP was less than observed for control LTP in naïve slices. We also examined lower concentrations of poly(I:C) and found that 4 hour incubation at 1 μg/ml blocked LTP (106.0 ± 6.6%, N = 5); however, AlloP again failed to prevent this LTP inhibition (99.0 ± 9.2%, N = 5; p = 0.5522).

### Allopregnanolone enantiomers modulate effects of LPS on learning *in vivo*

**Fig 7:**
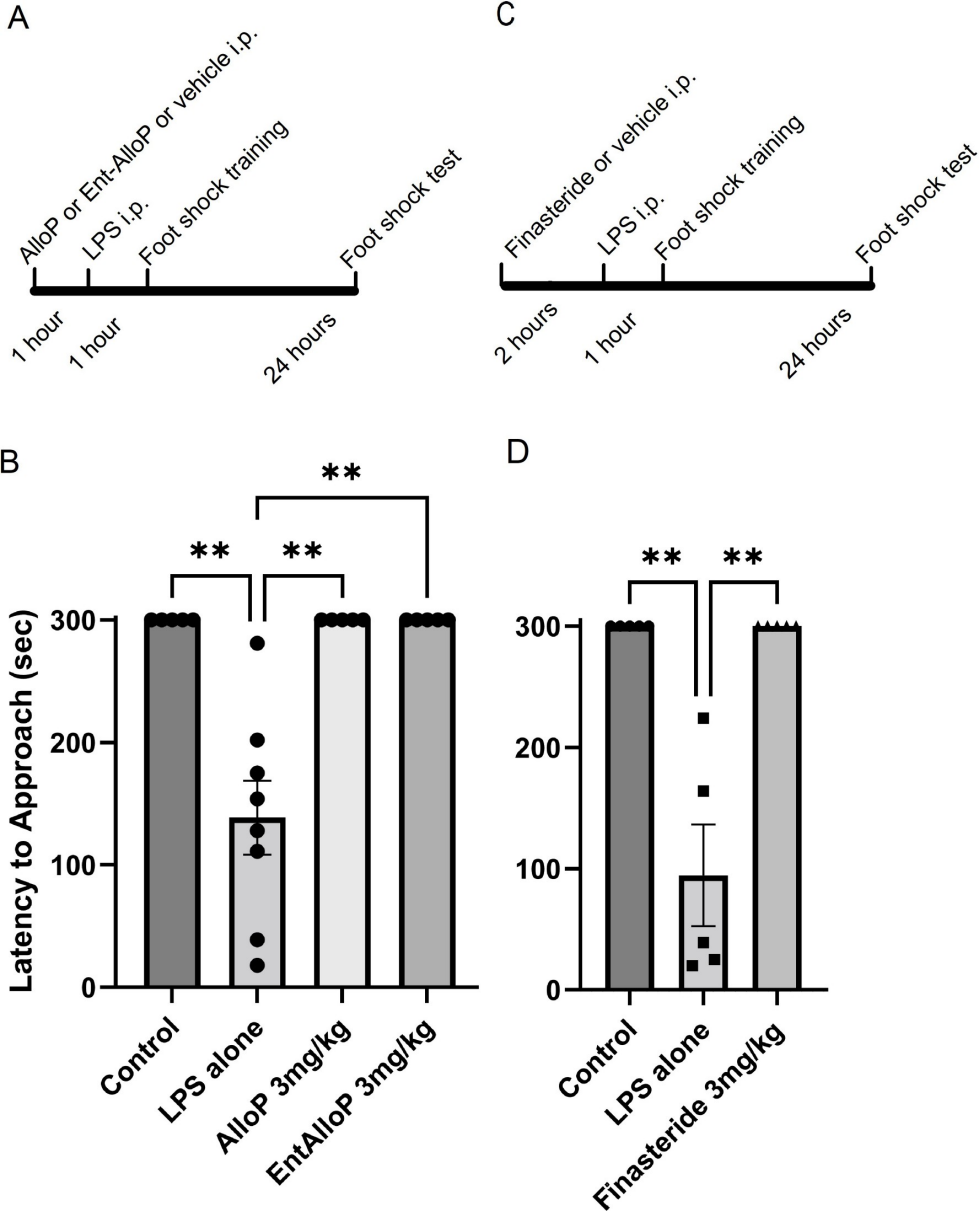
AlloP enantiomers and finasteride prevent the effects of LPS on one-trial inhibitory avoidance learning *in vivo*. Left graph: control rats, exposed to vehicle (DMSO) one hour prior to conditioning show robust learning 24 hours later and do not leave the lit chamber of the test apparatus. In contrast, animals pre-treated with vehicle followed by LPS (1 mg/kg ip) one hour later, show defective learning 24 hours following conditioning (p < 0.01 vs. controls). Effects of LPS on learning are completely prevented by pretreatment with 3 mg/kg (ip) AlloP or ent-AlloP one hour prior to LPS when tested 24 hours following conditioning. Right graph: finasteride pretreatment also prevented the effects of LPS on one-trial learning. The solvent for finasteride was 5% β-CDX (left bar). ** P < 0.01 by Dunn’s multiple comparison test.

To determine whether effects of the AlloP enantiomers observed above alter behavior *in vivo*, we examined the neuroactive steroids in a one-trial inhibitory avoidance learning task that is linked to CA1 hippocampal LTP [9, 18, 21]. For these studies, we focused on use of LPS because of its well-characterized acute effects on learning and memory and used a dose that minimized sickness behaviors based on prior experiments [9]. In the one-trial learning task, naïve rats readily associated entry from the lit chamber into the dark chamber with delivery of a foot shock, which they received one day prior to testing (Fig 7 left). These control animals remained in the lit compartment for the full 300 s testing period one day following training (N = 5). Rats treated with LPS (1mg/kg ip) one hour prior to training showed significant deficits in learning and remained in the lit chamber for only 138.5 ± 30.2 s (N = 8, Fig 7). Animals treated with either AlloP (3 mg/kg ip) or *ent*-AlloP (3 mg/kg ip) prior to training and LPS learned the task and performed the same as control rats (N = 5 each; Fig 7).

Based on our observations in *ex vivo* studies with the 5AR inhibitor, finasteride (Fig 3A), we also examined whether finasteride altered the effects of LPS on learning *in vivo*. Animals administered vehicle (5% CDX) again readily learned the task and remained in the lit chamber for the 300 s test trial. Rats treated with 1 mg/kg (ip) LPS showed dampened learning (latency to leave lit chamber: 94.4 ± 41.9 s, N = 5). At 3 mg/kg, finasteride completely inhibited the effects of LPS on one-trial learning (Fig 7 right).

## Discussion

Neuroinflammation contributes to multiple neuropsychiatric illnesses and to illness-related disability including cognitive dysfunction [1–4]. Thus, understanding the effects of neuroinflammation and its modulation provides an avenue to more effective treatments. In our prior studies, we examined acute effects of LPS, a bacterial endotoxin that evokes strong pro-inflammatory responses, using 10–15 min exposure to 1–10 μg/ml LPS. This LPS exposure rapidly and persistently dampens induction of LTP in the CA1 hippocampal region [9]. Surprisingly, the effects of μg/ml LPS did not require TLR4, a known LPS receptor [11–12], but appeared to involve non-canonical inflammasome signaling [6, 7, 48 but see 8]. Lower (ng/ml) concentrations of LPS also inhibited LTP but required longer (2–4 hour) exposures. In the present studies, we focused primarily on low concentrations of LPS to determine the role of TLR4 in its effects.

Our present results demonstrate that adverse effects of low LPS on LTP require TLR4 activation based on inhibition by two selective TLR4 antagonists. This LTP inhibition is also prevented by LPS-RS, a penta-acetylated form of LPS that inhibits both TLR4 and non-canonical inflammasome signaling [5, 25]. Akin to high concentrations of LPS, effects of low (ng/ml) LPS also involve local synthesis of 25-HC, an oxysterol inflammatory modulator [10, 49, 50]. In brain, 25-HC is largely, if not exclusively produced by microglia, and is markedly upregulated by LPS [9, 29, 31]. As in our earlier study [9], control LTP induction was unaffected by absence of Ch25H, the predominant synthetic enzyme for 25-HC, while adverse effects of low LPS concentrations on LTP were completely prevented. These results indicate that despite differences in the requirement for TLR4, both low and high LPS concentrations engage overlapping signaling to disrupt synaptic plasticity.

In our prior study, we found that high LPS disrupts CA1 LTP by promoting NMDAR-dependent metaplasticity [9]. In studies with other cellular stressors that activate similar metaplasticity, we found that the endogenous neurosteroid, AlloP, a potent and effective positive allosteric modulator (PAM) of GABA_A_ receptors, plays a key role in mediating acute LTP inhibition [13, 34]. Consistent with these observations, inhibiting AlloP synthesis with finasteride, a 5AR antagonist, prevents the effects of high (μg/ml) LPS on LTP. Finasteride also blocked the adverse effects of systemic LPS on one-trial inhibitory avoidance learning *in vivo*. In pilot experiments, we attempted to examine the effects of 5AR inhibitors against low LPS concentrations in hippocampal slices but found that long exposures to finasteride (2–4 hours) disrupted LTP in the absence of LPS.

As noted above, prior studies indicate that neurosteroids such as AlloP can mediate acute effects of cellular stressors on LTP [13, 34]. However, the role of 5-alpha neurosteroids in these acute adverse effects contrasts with other studies indicating that AlloP can dampen pro-inflammatory signaling [51], including effects mediated by TLR4 and certain other TLRs [14, 15]. These observations prompted us to examine whether AlloP could modulate and possibly prevent the effects of low (ng/ml) LPS on LTP. Exogenous AlloP prevented the effects of low LPS concentrations on LTP induction when administered just prior to and during LPS administration. In contrast to effects on low (ng/ml) LPS, we found that LTP inhibition by high (μg/ml) LPS was largely unaffected by AlloP, consistent with the importance of TLR4 in the effects of low but not high LPS concentrations. Furthermore, exogenous AlloP also prevented LPS-induced learning defects in a form of one-trial inhibitory avoidance learning that has been associated with CA1 LTP [19–21].

Signaling via TLR4 from the plasma membrane uses the adapter protein MyD88 [40], and prior studies found that exogenous AlloP dampens inflammatory signaling by TLR4 and other TLRs that act via MyD88 in a macrophage cell line [14, 15]. In contrast, AlloP did not alter responses mediated by TLR3, a pattern recognition receptor that signals from endosomes via MyD88-independent adapters [14, 15]. In our studies, agonists for multiple TLRs inhibited CA1 LTP when administered for 2–4 hours prior to tetanic stimulation. These included both MyD88-dependent (TLR2, TLR4 and TLR7) and MyD88-independent (TLR3) receptors. Exogenous AlloP prevented the adverse effects of MyD88-dependent receptors, but not TLR3 on LTP induction. TLR2 and TLR4 can signal from both the plasma membrane and from endosomes, and endosomal signaling engages the adapter proteins TRIF and TRAM [45, 46, 52, 53]. AlloP also promotes degradation of TLR2 and the adapter protein TIRAP, which is important for MyD88-signaling from cell surface by both TLR2 and TLR4 [16]. Thus, inhibition of TLR2 and TLR4 by AlloP may preferentially affect cell surface signaling by these TLRs. In contrast to TLR2 and TLR4, TLR3 and TLR7 signal exclusively from endosomes with TLR3 acting via TRIF [12, 54] and TLR7 acting via MyD88 [42]. Consistent with Balan and colleagues [15], we found that effects of TLR7 but not TLR3 were prevented by AlloP.

AlloP’s ability to modulate GABA_A_ receptors shows high enantioselectivity [22, 36], and GABAergic effects of AlloP contribute to its role in mediating the effects of stressors on hippocampal plasticity [13, 19] and its actions as a neuroprotectant [37, 38, 55]. Enantioselectivity of AlloP on GABA_A_ receptors has been shown in native hippocampal neurons, rat brain membranes and transfected cells expressing several GABA_A_ receptor subtypes [22, 36]. However, AlloP’s unnatural enantiomer also has anti-inflammatory [39] and neuroprotective properties [38, 39] that are independent of GABA_A_ receptors. The AlloP enantiomers accumulate identically in cell membranes and intracellular compartments [56–58]. Non-GABA actions that could contribute to anti-inflammatory and neuroprotective properties include stimulation of pregnane X receptors [39] and macroautophagy [38]. These observations prompted us to examine whether *ent*-AlloP also modulates TLR signaling. We found that *ent*-AlloP had variable effects against TLR4-mediated LTP inhibition but was highly effective against TLR2 and TLR7, and in the case of TLR2 had greater effects than AlloP itself. Despite variable effects against LPS in *ex vivo* LTP experiments, *ent*-AlloP was highly effective in preventing the adverse effects of LPS on learning and memory *in vivo*. Thus, the variable effects on LTP coupled with alternative mechanisms outlined above could have contributed to the overall protective effects of *ent*-AlloP on learning. In contrast to AlloP, which had no effect against TLR3, *ent*-AlloP significantly reduced LTP inhibition by TLR3, although LTP was not restored to control levels. Mechanisms by which the AlloP enantiomers modulate TLR signaling require further study, but differential effects on cell surface vs. endosomal signaling as well as actions on different TLR adapter proteins may be important.

**Fig 8:**
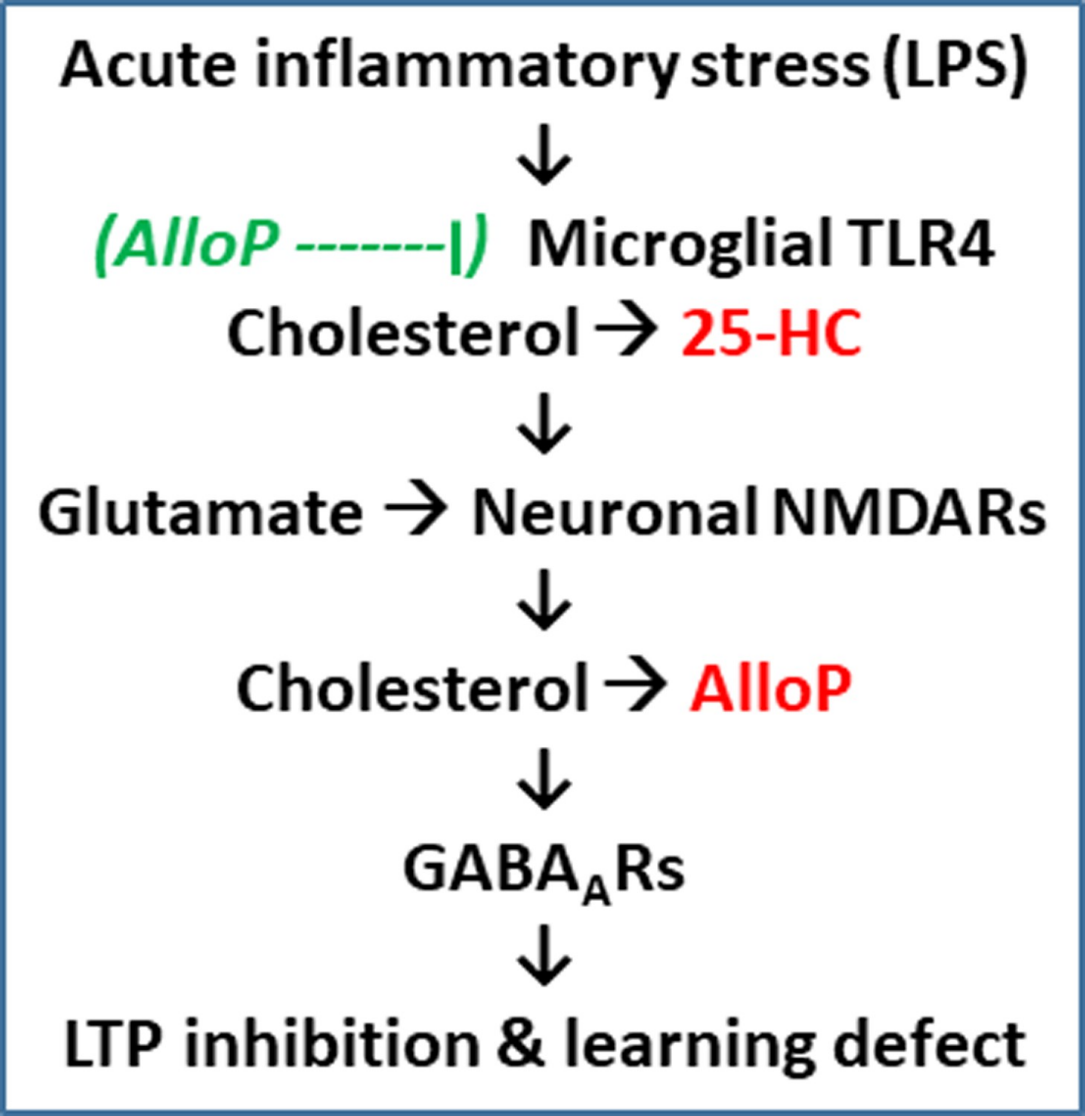
The diagram depicts mechanisms involved in LPS-induced LTP inhibition, highlighting the roles of AlloP as a mediator and a modulator of changes in synaptic plasticity. 25-HC and AlloP are highlighted in red as endogenous mediators of LTP inhibition. Text in green depicts the step at which exogenous AlloP appears to prevent the effects of LPS.

Results presented here indicate that 5-alpha reduced neurosteroids play complex roles in both mediating and modulating neuroinflammation (Fig 8). On the one hand, these steroids acutely mediate adverse effects of pro-inflammatory stimulation on hippocampal function and learning as evidenced by effects of 5AR inhibition. This result is consistent with prior observations in other forms of neuronal stress that evoke NMDAR-dependent LTP inhibition including ethanol, acetaldehyde, corticosterone and low concentrations of NMDA itself [13, 34]. In contrast, when exogenous AlloP is administered at a pharmacological dose just prior to and during low LPS, it prevents the adverse effects on CA1 LTP and hippocampal-dependent learning. This protective, modulating effect of AlloP extends beyond TLR4 to TLR2 and TLR7. Interestingly, neither exogenous AlloP nor *ent*-AlloP protects against high (μg/ml) LPS that inhibits CA1 LTP through a TLR4-indendent mechanism. However, we recently found that certain selective serotonin reuptake inhibitors (SSRIs), particularly fluvoxamine, can prevent the effects of high LPS concentrations by a mechanism that involves local neurosteroid synthesis and agonism at sigma 1 receptors [59].

## Conclusions

Taken together, these studies have implications for clinical use of neurosteroids and indicate that these agents have effects on pro-inflammatory signaling that could contribute to their clinical efficacy in neuropsychiatric illnesses [33]. Our present results also have implications for the possible development of neurosteroid enantiomers as therapeutics, given anti-inflammatory and neuroprotective actions observed in this and other preclinical studies [36].
